# Comparative study on factors affecting anaerobic digestion of agricultural vegetal residues

**DOI:** 10.1186/1754-6834-5-39

**Published:** 2012-06-06

**Authors:** Adrian Eugen Cioabla, Ioana Ionel, Gabriela-Alina Dumitrel, Francisc Popescu

**Affiliations:** 1 Faculty of Mechanical Engineering, University “Politehnica” of Timisoara, Bv. Mihai Viteazu no.1, Timisoara, 300222, RO; 2 Faculty of Industrial Chemistry and Environmental Engineering, University “Politehnica” from Timisoara, P-ta Victoriei no.2, Timisoara, 300006, RO

## Abstract

**Background:**

Presently, different studies are conducted related to the topic of biomass potential to generate through anaerobic fermentation process alternative fuels supposed to support the existing fossil fuel resources, which are more and more needed, in quantity, but also in quality of so called green energy. The present study focuses on depicting an optional way of capitalizing agricultural biomass residues using anaerobic fermentation in order to obtain biogas with satisfactory characteristics.. The research is based on wheat bran and a mix of damaged ground grains substrates for biogas production.

**Results:**

The information and conclusions delivered offer results covering the general characteristics of biomass used , the process parameters with direct impact over the biogas production (temperature regime, pH values) and the daily biogas production for each batch relative to the used material.

**Conclusions:**

All conclusions are based on processing of monitoring process results , with accent on temperature and pH influence on the daily biogas production for the two batches. The main conclusion underlines the fact that the mixture batch produces a larger quantity of biogas, using approximately the same process conditions and input, in comparison to alone analyzed probes, indicating thus a higher potential for the biogas production than the wheat bran substrate.

Adrian Eugen Cioabla, Ioana Ionel, Gabriela-Alina Dumitrel and Francisc Popescu contributed equally to this work

## Background

Anaerobic digestion (AD) is the natural process in which complex organic materials are broken down into simpler compounds in the absence of oxygen by the action of several micro-organism communities. Anaerobic digestion consists of four biochemical steps: hydrolysis - hydrolytic bacteria remove polymers to monomers; acidogenesis - acidogenic bacteria remove monomers to short carboxylic acid, CO_2_, hydrogen and alcohol; acetogenesis - the products of the previous phase are removed to acetic acid; methanogenesis - methane is built of the acetic acid [1-4].

The most important environmental benefit of the anaerobic digestion process is the production of biogas, a renewable energy source, which can be used as fuel for the internal combustion engines, for direct heating and, under better efficiency, in cogeneration, for electricity production as well [5]. The production of biogas based on biomass generates the reduction of fossil fuel use and enables the lowering of CO_2_-levels with fossil C origin, in accordance with EU directives regarding the climate changes and supporting the reduction of the green house gases emission especially, not mentioning the use of a local energy resource. Apart from yield of biogas, anaerobic digestion creates solid and liquid by-products, which can have value as a fertilizer or soil amendment.

The biogas produced by anaerobic digestion is a blend consisting mainly of methane (CH_4_ ≈ 60% by volume), carbon dioxide (CO_2_ ≈ 40% by volume), and small traces of hydrogen sulphide (H_2_S), hydrogen (H_2_), nitrogen (N_2_), carbon monoxide (CO), oxygen (O_2_), water vapor (H_2_O) or other gases and vapors of various organic compounds.

Due to the complexity of the bioconversion processes, many factors affecting the performances of an anaerobic digester were analyzed and depicted [6,7]. These can be divided in three main classes: (i) feedstock characteristics, (ii) reactor design and (iii) operational conditions. Among the operational conditions, temperature and pH are the most important parameters, thus the research was directed especially to these.

Anaerobic digestion is strongly affected by temperature [8,9]. Optimum temperature of mesophilic digester for biogas production is 35°C. In the mesophilic range, the activity and growth rate of bacteria decrease by 50% for each 10°C drop. Fall in biogas production starts, when temperatures decreases to 20°C and the production even stops at 10°C [1]. Increasing the temperature level up to 37°C leads to the time reduction required for the digestion process. Further increase in temperature decreases the rate of biogas generation.

The pH of the anaerobic digestion process is another parameter that has a significant effect on the digestion process [10-12]. The optimum pH range in an anaerobic digester is 6.8 to 7.2. However, the process can tolerate a range of 6.5 up to 8.0.

In the present paper, experimental investigation and results for anaerobic digestion of wheat bran and mix damaged ground grains in batch process have been reported.

## Results

### Substrates

**Figure 1:**
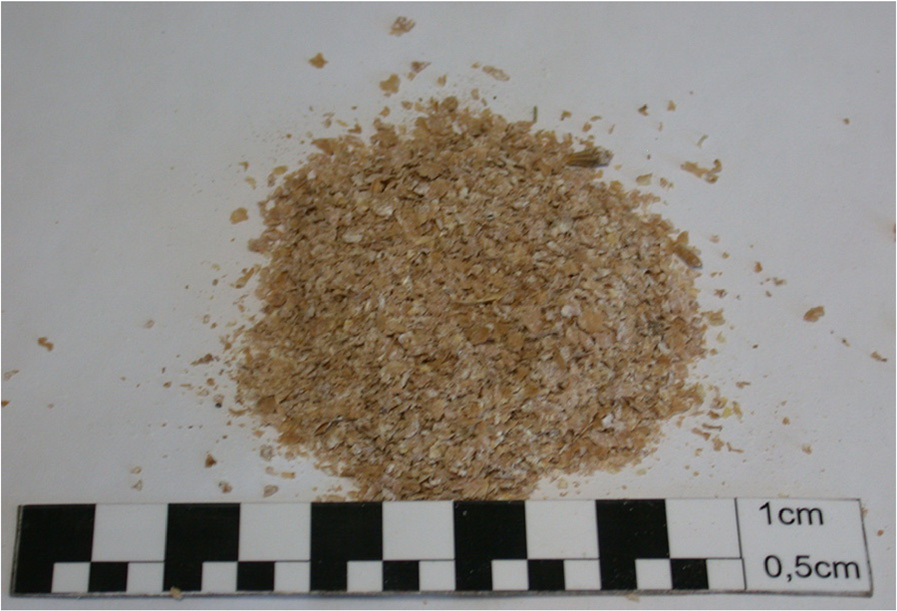
Wheat bran.

**Figure 2:**
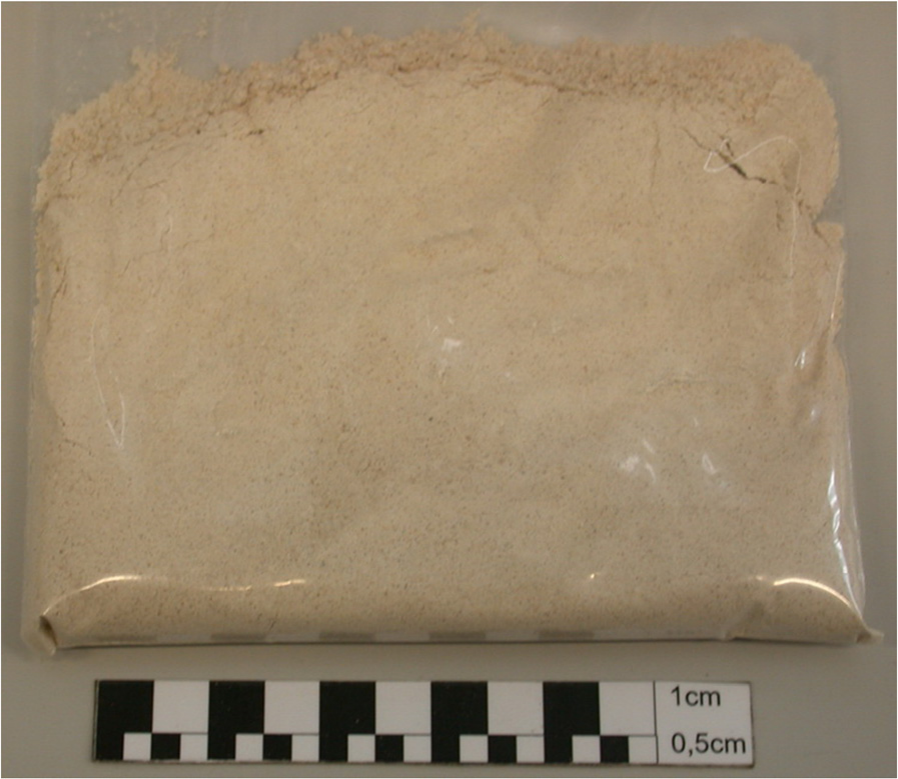
**Mix damaged ground grains.** 50% wheat and 50% corn.

Wheat bran and a mix of damaged ground grains (50% by mass wheat and the rest corn kernels) were used as substrates (Figure 1 and 2). The preparation of substrate was made according to Standard EN 14780 – Methods of preparing biomass samples.

The general characteristics of these substrates before and after the process are given in Table 1. These characteristics were obtained according to Standard EN 14774 – Determination of moisture content – oven dry method; Standard EN 14775, – Determination of ash content; Standard EN 14918, - Determination of calorific value. The substrates were stored at room temperature until further use.

**Table 1:** Main feedstock parameters at the beginning and the end of the process

**No.**	**Substrates**	**Humidity [%]**	**Ash content [%]**	**Lower heating value [kJ/kg]**	**Higher heating value [kJ/kg]**	**C/N**
Beginning of the process						
1	Wheat bran	10.23	4.63	15.535	17.098	88.3
2	Mix damaged ground grains (50% wheat rest corn kernels)	10.98	1.64	15.245	16.591	59.1
End of the process						
3	Wheat bran	0.29	15.14	7.356	6.094	-
4	Mix damaged ground grains (50% wheat rest corn kernels)	0.93	49.89	11.502	10.676	-

The chemical composition of the used substrate is presented in Tables 2 and 3. Determination of major and minor elements was achieved according to Standard EN 15290 and Standard EN 15297. Total C, H and N were determined according to EN 15104.

**Table 2:** Chemical composition of the used substrates

**No.**	**Chemical compound**	**Concentration in wheat bran [mg / kg]**	**Concentration in mix [mg / kg]**
1	Mg	1331	797
2	Al	71	55
3	Si	174	-
4	P	5855	2332
5	S	1165	1181
6	Cl	370	680
7	K	9697	4491
8	Ca	1209	716
9	Mn	108	31
10	Fe	177	81
11	Zn	69	23

**Table 3:** Heavy metals concentration in the used substrates

**No.**	**Heavy metal**	**Heavy metal concentration in wheat bran, [mg / kg]**	**Heavy metal concentration in the mix, [mg / kg]**
1	Cr	0.919	0.705
2	Mn	184.127	59.158
3	Co	1.385	0.6
4	Ni	1.494	-
5	Cu	6.053	-
6	As	0.366	-
7	Se	0.833	0.289
8	Br	5.472	10.114
9	Sr	7.256	2.098
10	Cd	4.102	3.58
11	Sn	-	0.584
12	Hg	-	-
13	Pb	6318	8.291

### Description of pilot plant

**Figure 3:**
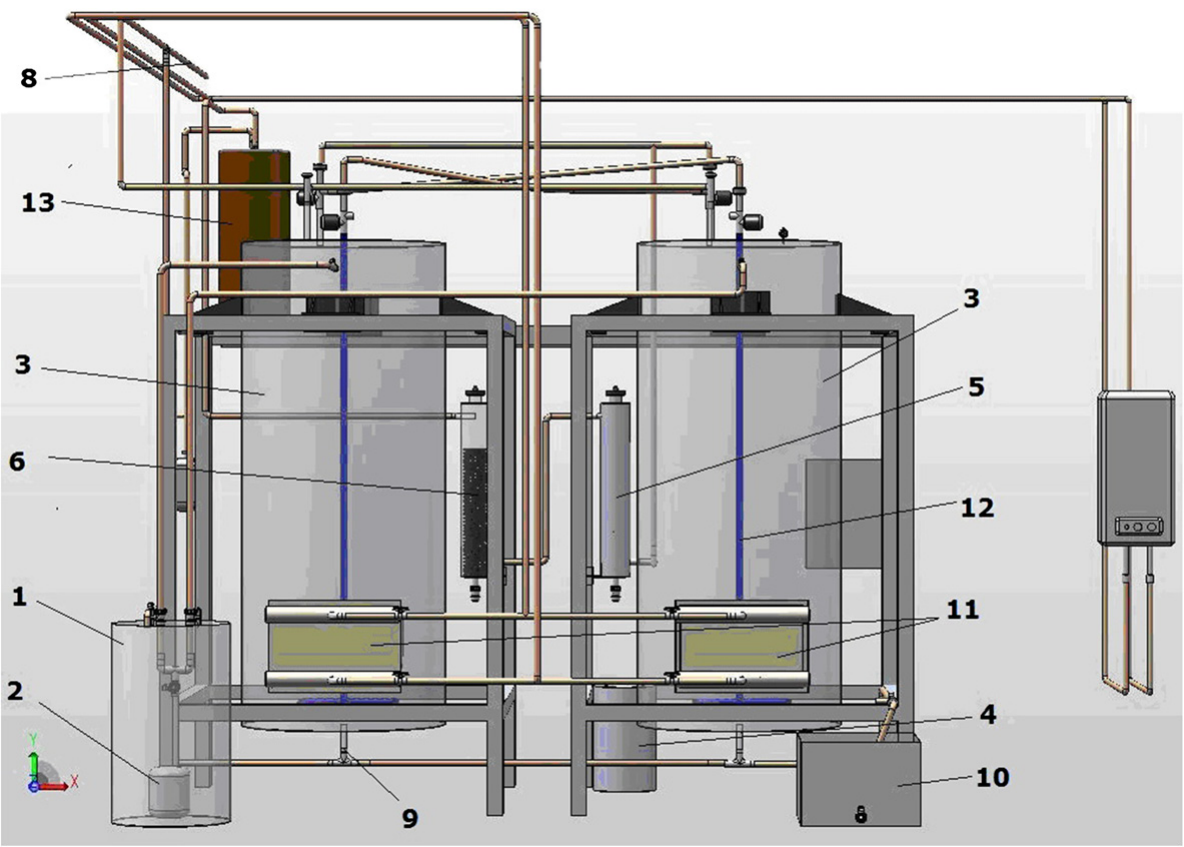
Schematic configuration of the pilot plant.

Figure 3 presents the pilot plant used for the biogas production through biomass anaerobic digestion. From the biomass deposit, the input material is passed through a mill, and then it is sent to the tank where the preparation of the suspension of biomass is occurring (1). The biomass suspension is transported with the help of the pump (2) and introduced into the fermentation reactors (3). The tank that feeds the agent necessary for the correction of the pH value assures, through the control system, the best conditions for an anaerobic fermentation process. The resulted biogas is passed further through a filter for the partially retaining the H_2_S (5) and after that, through a similar system used for the CO_2_ removal (6). In the next steps, in an adjacent system a CO_2_ desorption and compression occur. Finally the purified biogas is ready to be used and sent to the consumer (8). The used material is discharged by means of a gravimetric system (9) and the solid material is retained for being dried through a natural process. Next stage is the storage in a compost deposit, for being used as a soil fertilizer. A part of the resulting liquid is neutralized in the system (10), if necessary, and sent to the sewerage network. Another possibility consists of transporting it with the recirculation pump (2) from the suspension preparation tank (1). The fermentation reactors are thermostated, beeing heated with the system (11). For the homogenization of the suspension a bubbling system (12) made by polypropylene pipes to avoid the possible corrosion, is used. For depositing small quantities of biogas in the purpose of analyzing, the pilot is equipped with a small tank (13) positioned at the top of the reservoirs.

The reactors are fed at the beginning of the experiment with approximately 75 kg dry biomass and 2000 L water. The biogas production was measured daily, as well the pressure difference based on a pressure drop, using the semi-automated system and a gas counter. Methane (CH_4_) and carbon dioxide (CO_2_) compositions (v/v) were measured using a Delta 1600 IV gas analyzer. Temperature and pH were also continuously recorded.

## Discussion

**Figure 4:**
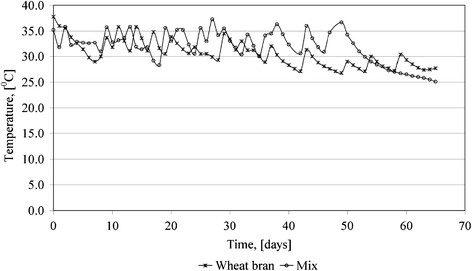
Temperature variation during anaerobic digestion process.

The variation of temperature during the anaerobic digestion process of two studied substrates is presented in Figure 4. One can observe that the temperature average value is around 31 – 32°C, with peaks at 36 – 37°C. The general behavior is connected with a combined regime: mesophilic regime for the first 40 days, for the wheat bran batch, and 50 days for the mix batch, including cryophilic regime for the last part of the process.

**Figure 5:**
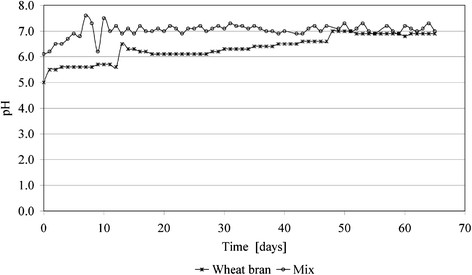
pH variation during anaerobic digestion process.

In addition to temperature, the pH is an important process parameter for the management of the biogas processes. The pH variation during anaerobic digestion process is presented in Figure 5. For wheat bran, the initial pH values are lower than for the mix batch and a general tendency of increasing from values of 6.5 up to 7 – 7.1 after 40 days is confirmed. In comparison, the behavior for the mix batch is much more linear, starting from pH values of 6, with small peaks inside the first 12 days, and stabilizing near the domain of 6.8 – 7.1, for the rest of the process. This indicates a better behavior of the mix during anaerobic fermentation, and has as advantage to be much easier controlled than for the first batch of material.

**Figure 6:**
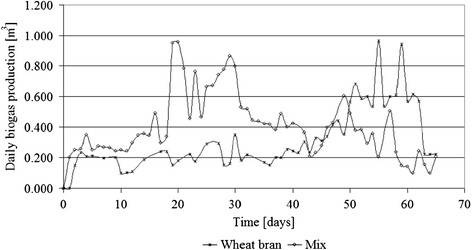
Daily biogas production during anaerobic digestion process.

From Figure 6 it can be observed that the daily biogas production underlines the fact that the mix batch is able to produce larger quantities of biogas, with average value of 0.405 m^3^/day, while the wheat bran batch had smaller average value of 0.323 m^3^/day. The obtained volumes are considered at normal pressure and the temperature existent inside the anaerobic tanks.

**Figure 7:**
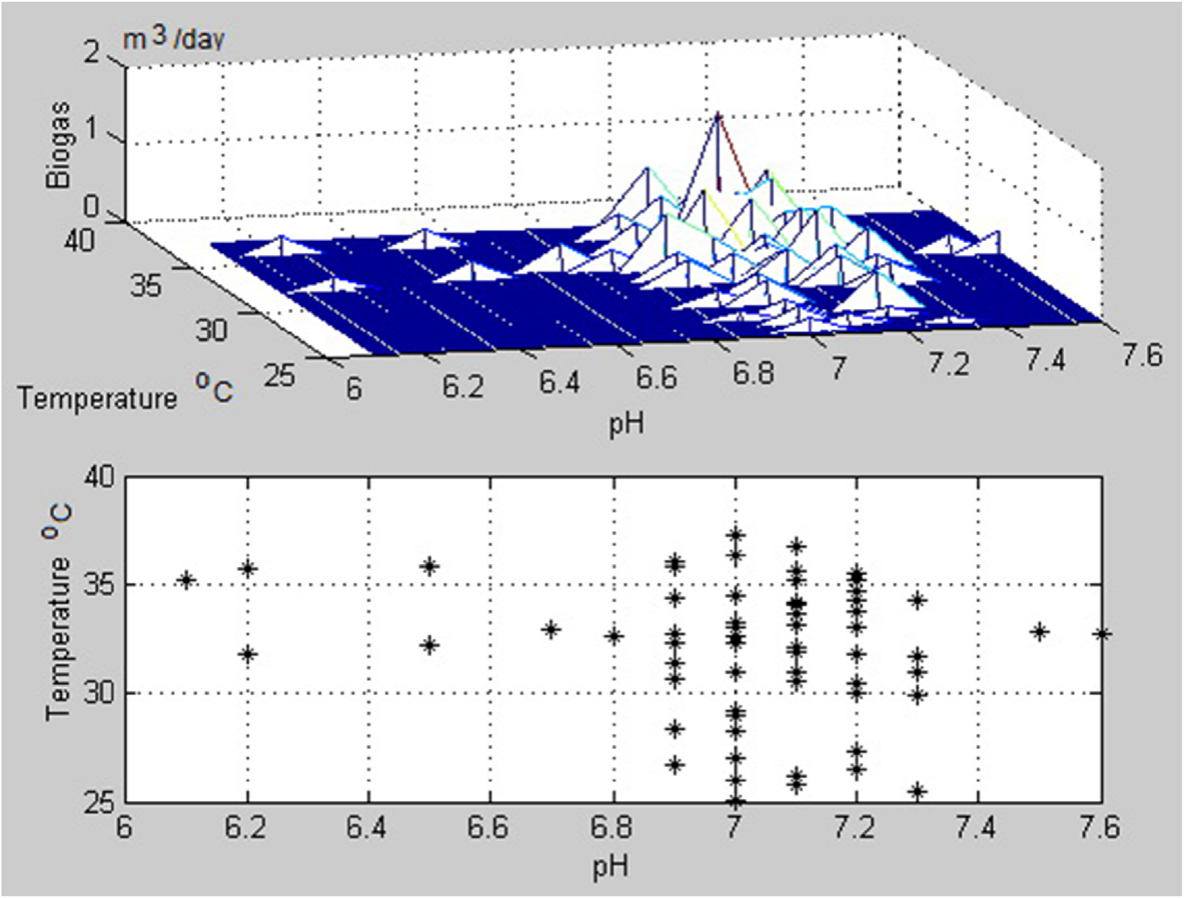
Cluster of the temperature and pH values for the mix and histogram for the produced consequent biogas amounts, in m3/day.

**Figure 8:**
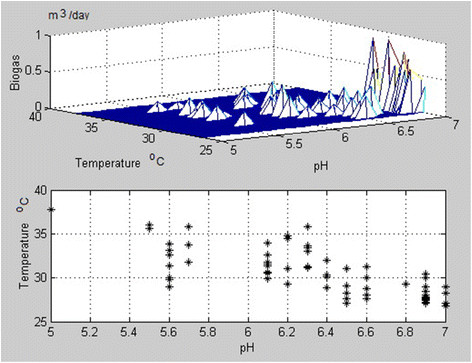
**Cluster of the temperature and pH values for the wheat bran and histogram for the produced consequent biogas amounts, in m**^**3**^**/day.**

By using the MATLAB software, the experimental data were processed and analyzed. The clusters of the temperature and pH values for mix and wheat bran batches and the histograms for the produced consequent biogas amounts are presented in Figures 7 and 8. The existing data can be used to determine the biogas quantities produced by each substrate for a known anaerobic digestion temperature and pH [13].

From Figure 7, it can be noticed that the higher value of biogas yield in case of an anaerobic digestion of mix is 0.955 m^3^ and correspond to a pH of 7 and a temperature of 33°C. The mix experiments were based on values measured during 65 days.

The correlation coefficient between T and the biogas volume generated during the anaerobic digestion (T-temperature in degree C and Q - amount of biogas in m^3^) (Figure 7) is 0.4642 that represents not a very large value, but a significant one, according to [14], where three domains −1 to −0.33; -0.33 to 0.33; and 0.33 to 1; are given. The correlation coefficient between pH and Q is 0.2737. This result is insignificant but still a positive value. The two quantities are still positively correlated meaning that the growth of one trains the increase of the other. These values are well corresponding to the location of the maxima in the cluster plan for the biogas production (Figure 7).

In correspondence, Figure 8 indicates that the highest yields of biogas for anaerobic digestion of wheat bran is 0.963 m^3^/day and corresponds to a pH of 6.9 and a temperature of 29°C .

The anaerobic digestion of this batch was monitored during 65 days, also.

The correlation coefficient between T and Q (temperature and amount of biogas) is - 0.508, and is considered as a significant one. The coefficient between the pH and Q value is 0.6892, and is also considered significant. The two quantities are positively correlated, the correlation coefficients corresponding to the location of the maxima in the cluster plan of the biogas production. The two parameters are positively correlated, indicating clearly that the growth of one trains the increase of the other.

## Conclusions

The presented study underlines the potential of using different degraded cereal biomass in order to obtain biogas using the anaerobic fermentation process.

Based on the two series of experiments and results, the mix of wheat and corn kernels proved to be more suited for biogas production than the wheat bran batch, for both one considering the general parameter variation in time and the produced biogas quantities.

**Figure 9:**
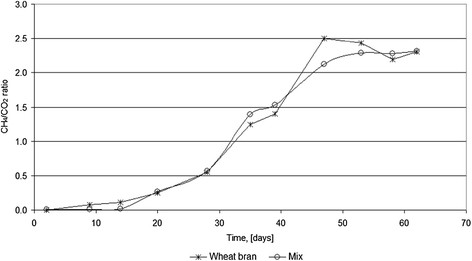
Obtained CH4/CO2 ratios for wheat bran and mix damaged ground grains.

The total volume of biogas produced during the anaerobic digestion process was 17.8 m^3^ for wheat bran substrate and 25.1 m^3^ for mix substrate respectively. The maximum methane and CO_2_ concentrations (by volume) inside the produced biogas were 68% methane and 32% CO_2_ for the mixture batch and 69% methane and 31% CO_2_ for the wheat bran batch. The CH_4_/CO_2_ ratios resulted are presented in Figure 9, for both substrates. The obtained values of correlation coefficients and the related histograms (Figures 7 and 8) demonstrate that the method is enough accurate to describe de production of biogas by anaerobic digestion as a function of process pH and temperature, while the physical values are still positively correlated meaning that the growth of one trains the increase of the other. The values (even different for both ranges related to studied materials) are in good similitude to the position of the corresponding location of the maxima in the cluster plan for the biogas production.

Similar correlation coefficients were obtained in a further reproducibility tests on same substrate leading to a conclusion that the model can be successfully used for this type of material in anaerobic digestion without inoculums and any further added dry biomass during the process. As other authors observed [15], ensiling dose does not increase the methane yield, for any crop materials.

Thus, one concludes that the described technology by using vegetal biomass is a relevant solution for using and thus solving the problem of the existing degraded materials which are not capitalized. By comparing two ranges of experiments, conclusions that a better solution is offered by mixtures, are drawn. The analysis accomplished between the experimental data of biogas produced, pH and temperature values supports the conclusions. The value of the technology proposed might be extended by using the resulted compost as fertilizer for agricultural crops.

## Methods

### Material preparation for the anaerobic fermentation

Both materials were prepared similar for the anaerobic fermentation process: the material was subject to dimension reduction with a Retsch SM2000 grinding device to a dimensions of 1 – 2 mm. The selected materials for the experiments were placed inside the anaerobe reactors through the means of a submersible pump, the ratio between the solid and liquid material being 75 kg to 2000 L. The internal agitation occurred by the means of a bubble system, inserted at the bottom of each reactor and by using as agitation factor a part of the produced biogas. The pH corrections were accomplished using a lime suspension with a correction of the pH-value of 12 – 13. The suspension was inserted inside the reactors by means of dosing pumps (Hanna Instruments, model BL20). The obtained biogas was analyzed with a Delta 1600 S – IV gas analyzer for CO_2_ and CH_4_ composition with an accuracy domain of +/−5% of reading both for CH_4_ and CO_2_.

### Laboratory analysis

*For the determination of moisture content*, the used equipments are: Sartorius AC211 laboratory balance with four decimal precision, weighing dishes, a desiccator and a drying oven (model DHG-9040, A Series). The substrates were systematically weighted with the balance before, during and after the drying process until stable mass. The period of time inside the drying oven was between 2 and 4 hours.

The used formula for the determination is:

(1) $$M_{\mathrm{ad}}=\frac{\left(m_{2}-m_{3}\right)}{\left(m_{2}-m_{1}\right)}\cdot ;100$$

*m*_*1*_ = is the mass in grams of the empty dish

*m*_*2*_ = is the mass in grams of the empty dish plus sample before drying

*m*_*3*_ = is the mass in grams of the empty dish plus sample after drying

At least three determinations for each material were achieved.

*For the determination of ash content* the used equipments are: Sartorius AC211 laboratory balance with four decimal precision, weighing dishes, a desiccator and a furnace (model L1206 – Caloris Group). The empty dishes were inserted inside the furnace at 815°C for a period of 2 – 3 hours. The materials were measured with the balance, put inside the empty dishes and inside the furnace for approximately 2 hours. After the process was finished, the materials were put near the furnace for 10 minutes to cool and then inside the desiccator for 10–15 minutes. After those steps, the materials are weighed again.

The proposed formula for the determination is:

(2) $$A_{d}=\frac{\left(m_{3}-m_{1}\right)}{\left(m_{2}-m_{1}\right)}\cdot 100\cdot \frac{100}{100-M_{\mathrm{ad}}}$$

*m*_*1*_ = is the mass in grams of the empty dish

*m*_*2*_ = is the mass in grams of the empty dish plus sample

*m*_*3*_ = is the mass in grams of the empty dish plus ash

*M*_*ad*_ is the% moisture content of the test sample used for determination.

Again at least three determinations for each material were carried out.

*For the determination of the calorific value* there was used a Sartorius 320 laboratory balance with four decimal precision, a calorimeter bomb model IKA C 5000, metal dishes for the bomb, cotton fuses, a pellet press, a ion chromatograph model Dionex IC 20, distilled water and glass bottles for the liquid samples. A quantity of about 0.7 grams of material was pressed inside the pellet press, weighed without the cotton fuse, and introduced inside the bomb. After approximately 40 minutes, the sample was removed from the bomb, washed with 100 ml distilled water and the registered value indicated by the apparatus is inserted into a protocol. The liquid sample was further analyzed inside the ion chromatograph for chlorine, sulphur and nitrates and the obtained values are used for correcting the initial values, together with hygroscopic humidity and ash content. Again, one mentions that at least three determinations for each material were made.

*Determining of major and minor elements* was based on a two step method. First, each sample was introduced inside a hot press, at a temperature level of 140°C and a force of 50 kN for a period of 330 sec. For the second step, the pressed materials were inserted into a MagiXPro X – Ray Fluorescence Spectrometer for a period of 20 minutes / sample for major elements and 1.5 hours / sample for the minor elements. The results were stored and imported via PC.

For the determination of C and N content the LECO TruSpec CHN analyzer was used, with dedicated software and a Sartorius 320 laboratory balance. Before the determination, a general analysis of the system was made, through blind tests and standard materials for equipment calibration. The obtained values were used for recalculation of the results up to their constancy and the average value was considered.

### Mathematical analysis

The correlation coefficient used for data analysis was developed based on real laboratory data, as resulted from the two anaerobic digestion experiments. By analyzing the set of experimental data, it was assumed that the dependence between the biogas production and pH and temperature of the substrates is best to be evaluated by means of the regression coefficient. Also the analysis by means of histograms between the biogas volumes (quantity) generated under different temperature and pH values was proposed.

In general agreement to the basic theory and the application developed in [14], was used the following general formula for the correlation coefficient:

(3) $$R=\frac{\frac{1}{N}\left[\sum_{i}x_{i}y_{i}-\sum_{i}x_{i}\sum_{i}y_{i}\right]}{\frac{1}{N}\sqrt{\left[\sum_{i}{\left(x_{i}-\frac{1}{N}\sum_{i}x_{i}\right)}^{2}\right]\left[\sum_{i}{\left(y_{i}-\frac{1}{N}\sum_{i}y_{i}\right)}^{2}\right]}}$$

where:

x_i,_ y_i_ with i = 1, 2, … N, are sample values of the measured quantities (physical values) for which the correlation coefficients are calculated. In particular they represent the substrate pH value during anaerobic digestion, as a function of produced biogas volume [m^3^/day] and the substrate temperature during anaerobic digestion process [°C] as a function of produced biogas volume [m^3^/day].

## Competing interests

II and FP are co-holders of the Romanian Patent no. 122047 related to biogas production method and pilot installation from biomass.

## Authors’ contributions

AEC, II and FP planed the activities. AEC and FP performed the experimental research and elaborate the paper. II coordinated the whole research including the drawing of the conclusions and coordinated the final form of the paper. GAD performed the mathematical approach and analyze. All authors read and approved the final manuscript.
